# Bipyrimidine Signatures as a Photoprotective Genome Strategy in G + C-rich Halophilic Archaea

**DOI:** 10.3390/life6030037

**Published:** 2016-09-02

**Authors:** Daniel L. Jones, Bonnie K. Baxter

**Affiliations:** Great Salt Lake Institute and Biology Program, Westminster College, Salt Lake City, UT 84105, USA; dlj0317@westminstercollege.edu

**Keywords:** halophilic archaea, DNA damage, DNA repair, photoprotection, cyclobutane pyrimidine dimer, bipyrimidine, genome evolution

## Abstract

Halophilic archaea experience high levels of ultraviolet (UV) light in their environments and demonstrate resistance to UV irradiation. DNA repair systems and carotenoids provide UV protection but do not account for the high resistance observed. Herein, we consider genomic signatures as an additional photoprotective strategy. The predominant forms of UV-induced DNA damage are cyclobutane pyrimidine dimers, most notoriously thymine dimers (T^Ts), which form at adjacent Ts. We tested whether the high G + C content seen in halophilic archaea serves a photoprotective function through limiting T nucleotides, and thus T^T lesions. However, this speculation overlooks the other bipyrimidine sequences, all of which capable of forming photolesions to varying degrees. Therefore, we designed a program to determine the frequencies of the four bipyrimidine pairs (5’ to 3’: TT, TC, CT, and CC) within genomes of halophilic archaea and four other randomized sample groups for comparison. The outputs for each sampled genome were weighted by the intrinsic photoreactivities of each dinucleotide pair. Statistical methods were employed to investigate intergroup differences. Our findings indicate that the UV-resistance seen in halophilic archaea can be attributed in part to a genomic strategy: high G + C content and the resulting bipyrimidine signature reduces the genomic photoreactivity.

## 1. Introduction

Halophilic microorganisms thrive in the briny waters of salt lakes and evaporative solar salterns [1]. Saturated salinity, from 25%–35%, presents osmotic obstacles for life, but we see representatives from all three domains thriving in these conditions [2]*.* Hypersaline habitats select for archaea at a higher abundance than bacteria and eukarya in the community [3]. The present study focuses on this particular group, which likely share genetic strategies for life at high salinity [2]. Microbial diversity studies show a wide array of halophilic archaea present in hypersaline ecosystems [4] that are adapted to thrive in the saltiest places on Earth.

Beyond salt, high solar radiation is a feature of these environments; thus, their microbial inhabitants must have evolved to overcome the challenge of ultraviolet (UV) exposure. Halophilic archaea are highly resistant to UV light, first noted by Dundas and Larsen [5]. For example, one *Halorubrum* species was previously found to be nearly ten-fold more UV-resistant than *Escherichia coli* [6]. This is due in part to their robust UV DNA repair systems, including photoreactivation and nucleotide excision repair [7,8,9,10,11], but there are clearly additional processes that afford the high level of resistance to solar radiation exhibited by halophilic archaea.

Several studies have proposed other potential photoprotective strategies; for example, carotenoids in the cell membranes of halophilic archaea may provide UV resistance [6,12,13], although the mechanism of which remains unclear. These organisms have a unique composition of carotenoids that set halophilic archaea apart [14,15,16,17]. In fact, some halophiles may have an array of genes that allow their complex interplay with light [18], including carotenoid biosynthetic pathways, and also genes encoding retinal-containing proteins like bacteriorhodopsin, or “purple membrane” protein, which pumps protons out of the cell upon exposure to light [19] and may aid in the generation of ATP. An environmental genomics approach to saltern communities found the presence of a large variation of rhodopsin-like genes [20], indicating the significance of photobiology for halophiles.

**Figure 1:**
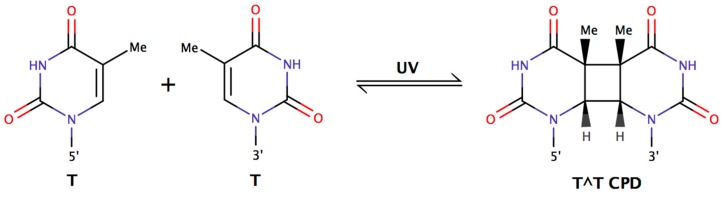
Thymine-thymine cyclobutane pyrimidine dimer (T^T CPD) forms via butane ring cyclization between adjacent thymines on the same strand of DNA. Similar chemistry occurs at the other bipyrimidine nucleotides TC, CT, and CC [27].

One photoprotective mechanism suggested in the literature for decades [22,23,24,25,26] relates to the genomes of organisms with high Guanine+Cytosine (G + C) content, such as halophilic archaea. Their genomes typically exceed 60% G + C (Table 1), which effectively reduces the number of thymines (Ts) present. Thymine dimers (T^T), which form from the UV-induced cyclization of two adjacent thymines on the same DNA strand (Figure 1), were long thought to be the primary DNA damage arising from sunlight [23]. Logically, the limitation of T residues is expected to reduce the incidence of adjacent Ts on the same DNA strand and thus, the possibility of T^Ts. Reduction of DNA damage would reduce mutagenesis during replication of unrepaired lesions. Kennedy et al. [26] suggested that this strategy of thymine limitation could explain the UV resistance of halophilic archaea, but the idea remains untested.

The most important oversight in this premise is that T^T lesions are but a subclass of cyclobutane pyrimidine dimers (CPDs), which include each possible adjacent bipyrimidine pair: (5’ to 3’) T^T, C^C, C^T, and T^C. Haynes [21] noted that the sensitivity of microorganisms to UV light was not mathematically proportional to the thymine frequency in the genome, which pointed to other lethal lesions. Any set of two adjacent pyrimidines may cyclize upon exposure to a photon of UV light. Though CPDs represent the majority of solar-induced DNA damage events [28], other potential lesions resulting from the UV-irradiation of bipyrimidine sequences are pyrimidine (6-4) pyrimidone photoproducts (6-4PPs). The proportion of CPDs to 6-4PPs is dependent on wavelength of light [29] and on flanking sequences [30,31].

Though it is logical to assume T^T lesions may be limited in halophilic archaea, other photolesions may form; therefore, the photoreactivities of each bipyrimidine pair and the relationship between G + C content and their incidences must be considered in order to assess any photoprotective benefit [32,33]. Therefore, we sought to address the hypothesis that thymine limitation is photoprotective using a genome photoreactivity score that incorporates all bipyrimidine pairs and their respective photoreactivities, while accounting for all potential types of UV damage (e.g. CPDs and 6-4PPs). We developed an equation to quantify the theoretical photoreactivity of a genome based on the frequencies of each bipyrimidine doublet within it, weighted by their intrinsic photoreactivities (as determined by [33]) and genome size. Comparison with other groups and a robust statistical analysis sheds light on an interesting story in genome evolution.

## 2. Materials and Methods

### 2.1. Comparing G + C Content of Halophilic Archaea Versus Other Prokaryotes

A list of all prokaryotes with full-genome sequences available (*n* = 5074) and their corresponding G + C contents was downloaded from the NCBI database [21]. One representative genome for each species was selected at random, yielding sample groups of *n* = 29 halophilic archaea and *n* = 2231 other prokaryotes.

### 2.2. Genome Sampling

Tabulated lists of all species with full-genome sequences available were downloaded from the NCBI database [21] for the sample groups bacteria (taxid: 2, *n* = 4829), halophilic archaea (taxid: 183963, *n* = 33), archaea excluding halophilic archaea (taxid: 2157, *n* = 209), cyanobacteria (taxid: 1117, *n* = 90) and enterobacteriaceae (taxid: 91347, *n* = 736).

Steps were taken to minimize sample bias: for the halophilic archaea group, one representative strain from each species was selected at random, yielding a sample group of *n* = 29 halophilic archaea. For the archaea, cyanobacteria, and enterobacteriaceae groups, one representative from each genus was selected at random, yielding sample groups of *n* = 68 (non-halophilic) archaea, *n* = 32 cyanobacteria, and *n* = 42 enterobacteriaceae. For the bacteria, the first 101 strains of a unique genus to be randomly selected constituted the final sample group of *n* = 101 bacteria.

The full-genome sequences corresponding to each sampled strain were downloaded as .fasta files from the NCBI database [21].

### 2.3. Determining Bipyrimidine Incidences

To determine the incidences (i.e., relative frequencies) of each bipyrimidine in the sampled genomes, a novel word-counting program “DinucleotideCounts” was written in the scripting language R (script available at: [34]). This program determines the frequency of each dinucleotide, the frequency of each nucleotide, and the size of any .fasta-formatted DNA sequence.

The bipyrimidine frequencies within sampled genomes were determined via the DinicleotideCounts program. Bipyrimidine incidences (TC_i_, TT_i_, CT_i_, CC_i_) were then computed by dividing each bipyrimidine’s frequency by the size of the corresponding genome in bases.

### 2.4. Determining Theoretical Genomic Photoreactivity (P_g_)

We devised the metric *P_g_* to quantify the theoretical photoreactivity of a genome based on its bipyrimidine signature: *P_g_* corresponds to the weighted sum of a genome’s bipyrimidine incidences (TC_i_, TT_i_, CT_i_, CC_i_):

*P_g_* = 1.73(TC_i_) + 1.19(TT_i_) + 0.61(CT_i_) + 0.39(CC_i_) (1)

The coefficients represent the intrinsic photoreactivity of each bipyrimidine, as experimentally determined by [33] via establishing the ratio between the frequency of the photoproducts (6-4PPs and CPDs) and the bipyrimidine incidences in DNA with varying G + C content.

### 2.5. Statistical Methods

**Figure 2:**
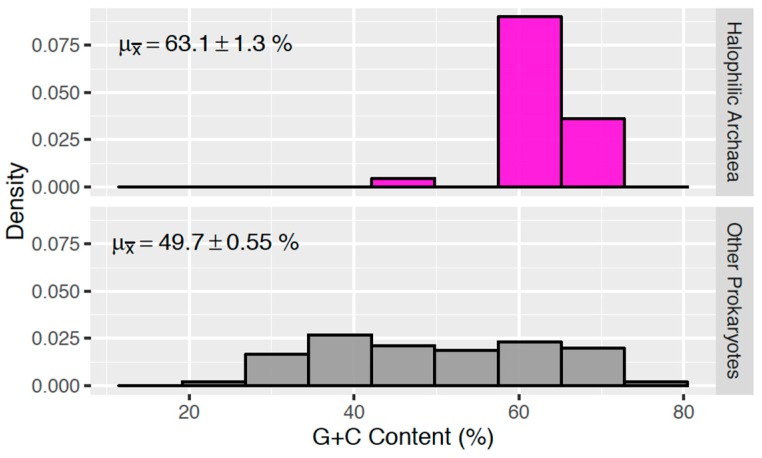
Genomic G + C content (%) distributions for samples of halophilic archaea (*n* = 29) and other prokaryotes (*n* = 2231). Sample means are denoted with +/− 1.96 standard errors. *p* < 2.2 × 10^−16^.

**Figure 3:**
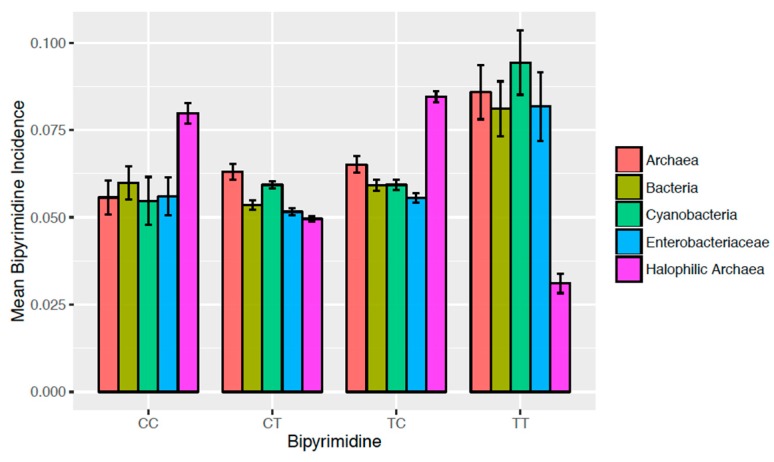
Mean bipyrimidine incidences for samples of (non-halophilic) archaea (*n* = 68), bacteria (*n* = 101), cyanobacteria (*n* = 32), enterobacteriaceae (*n* = 42), and halophilic archaea (*n* = 29). Error bars represent +/− 1.96 standard errors.

**Figure 4:**
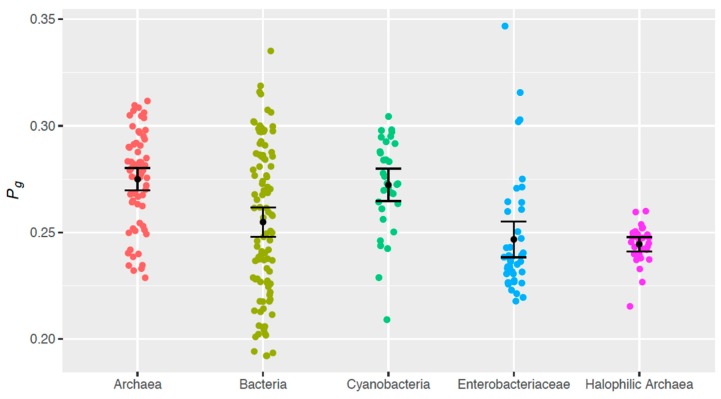
Distributions of the theoretical genomic photoreactivity *P_g_* computed for samples of (non-halophilic) archaea (*n* = 68), bacteria (*n* = 101), cyanobacteria (*n* = 32), enterobacteriaceae (*n* = 42), and halophilic archaea (*n* = 29). Sample means are marked with error bars representing +/− 1.96 standard errors.

**Figure 5:**
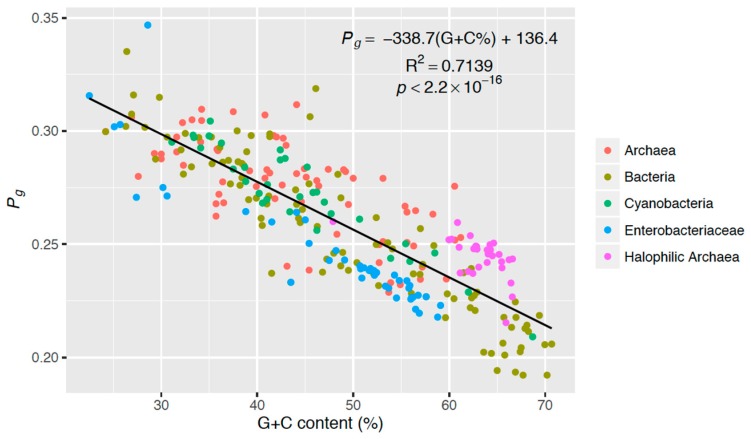
Theoretical genomic photoreactivity *P_g_* versus G + C content (%) of each sampled genome (*n* = 272). The genomes of the five taxonomic groups are indicated by color.

G + C content averages were compared (Figure 2) via a Welch Two Sample t-test using the “stats” package in R [35]. Intergroup differences in bipyrimidine incidences (Figure 3) and *P_g_* (Figure 4) were assessed via one-way analysis of variance (ANOVA) and post-hoc Tukey contrasts using the “multcomp” package [36] in R [35]. The regression analysis of G + C content vs. *P_g_* (Figure 5) and corresponding Pearson’s product-moment correlation test were carried out using the “stats” package in R. All randomization was facilitated by the “RAND” function in Microsoft Excel.

## 3. Results

### 3.1. G + C Content of Halophilic Archaea

Halophilic archaea are known to have high G + C content with one notable exception, *Haloquadratum walsbyi* [37,38,39]. To understand the unique genomic features of these halophiles, we compare them to other prokaryotes. The NCBI Genbank database contains the full genome sequences for 29 species of halophilic archaea (Table 1), which were utilized for this study. 2231 other prokaryotic species (bacteria and non-halophilic archaea) were selected as described in Materials and Methods. This analysis (Figure 2) showed that halophilic archaea (excluding *H. walsbyi*) have a clustered distribution with a remarkably high G + C content (63.1% ± 1.3%) relative to other microbial life (49.7% ± 0.55%), thereby demonstrating the uniqueness of this group and pointing to the relatedness of halophilic archaea.

### 3.2. Bipyrimidine Signature of Halophilic Archaea

Due to their high G + C content (Figure 2), we predicted halophilic archaea would have low and high incidences of TT and CC dinucleotides, respectively. However, considering the incidences of all bipyrimidine pairs (CC_i_, CT_i_, TC_i_, and TT_i_) is necessary for understanding the overall photoreactivity of their genomes. Hence, bipyrimidine incidences were determined for and subsequently compared between halophilic archaea and the other taxonomic groups bacteria, non-halophilic archaea, cyanobacteria, and enterobacteriaceae (*as described in Materials and Method*s) for insight into unique genomic signatures. If an evolutionary genome strategy were employed to protect organisms from sunlight, one would expect that a photosynthetic group like cyanobacteria might share such a strategy, and that enterobacteriaceae bacteria, which dwell inside higher eukaryotes, would not. Random samplings of species in these groups are thus included as controls, in addition to the bacteria and non-halophilic archaea (Figure 3).

As predicted, halophilic archaea are distinctive among these control groups in their low TT_i_ and high CC_i_ (Figure 3). Further, it was found that halophilic archaeal genomes are also characterized by high TC_i_ relative to the comparative groups. Multiple comparisons of means with respect to each bipyrimidine pair (*as described in Materials and Methods*) point to some interesting observations, indicating that on average:

- Halophilic archaea have larger CCi than any other group (*p* < 10^−4^ each). No other significant intergroup differences in CCi were detected.
- Halophilic archaea have smaller CTi than archaea and cyanobacteria (*p* < 10^−4^ each). Other significant differences in CTi were found between enterobacteriaceae vs. archaea (*p* < 10^−4^), cyanobacteria vs. bacteria (*p* = 2.35 × 10^−4^), and enterobacteriaceae vs. cyanobacteria (*p* < 10^−4^).
- Halophilic archaea have larger TCi than any other group (*p* < 10^−4^ each). Archaea have the next highest level of TCi, being larger than bacteria (*p* < 10^−4^), cyanobacteria (*p* = 3.27 × 10^−3^), and enterobacteriaceae (*p* < 10^−4^).
- Halophilic archaea have smaller TTi than any other group (*p* < 10^−4^ each). No other significant intergroup differences in TTi were detected.

### 3.3. Intergroup Differences in Theoretical Genomic Photoreactivity (P_g_)

Until recently, TTs were thought to be the most photoreactive sequences [24]. This idea has been challenged by current data on the photoreactivity of each bipyrimidine pair in both naked and intracellular DNA with a variety of genome G + C contents [31,32]. Matallana-Surget et al. [33] experimentally found the relative photoreactivities of the bipyrimidine sequences to be in the decreasing order of TC > TT > CT > CC. Furthermore, these authors quantified the intrinsic photoreactivity of each bipyrimidine via determining ratio between frequency of photoproducts (CPDs and 6-4PPs) and bipyrimidine incidences in DNA with varying G + C content. These ratios were employed as coefficients in the equation for our theoretical quantification of genomic photoreactivity in terms of bipyrimidine signature, *P_g_*, (Equation (1)). *P_g_* was calculated for each sampled genome and then compared across all tested taxonomic groups (Figure 4).

Intergroup differences in *P_g_* were examined via a multiple comparison of means (*as described in Materials and Methods*), which indicated that on average:

- Halophilic archaeal genomes have a smaller *P_g_* than do genomes from (non-halophilic) archaea (*p* < 10^−3^) and cyanobacteria (*p* = 1.01 × 10^−3^).
- Similarly, enterobacteriaceae genomes are less photoreactive than (non-halophilic) archaea (*p* < 10^−3^) and cyanobacteria (*p* = 1.02 × 10^−3^).
- (Non-halophilic) archaeal genomes have a larger *P_g_* than do genomes from the other sample groups, with the exception of cyanobacteria (*p* < 10^−3^).

### 3.4. Genomic Strategy of Photoprotection

In analyzing photoreactivity in the studied genomes, we plotted each computed *P_g_* value against its corresponding genome’s G + C content (Figure 5). These data clearly show a strong, negative correlation between *P_g_* and G + C content (R^2^ = 0.7139, *p* < 2.2 × 10^−16^). Figure 5 further demonstrates that this relationship holds true not only for the halophilic archaea tested, but also for all other genomes with this bias toward G + C, altogether giving evidence that an organism’s genomic G + C content contributes to their relative sensitivity to UV radiation.

## 4. Discussion

Solar exposure for all prokaryotic life on Earth results in UV-induced DNA damage, the majority of which is in the form of CPDs, a cycloaddition of two adjacent pyrimidine bases (Figure 1) [28,31]. CPDs are mitigated by two universal DNA repair systems: photolyase [40,41] and nucleotide excision repair [42]. Halophilic archaea are no exception as they have a similar ratio of CPD to 6, 4 photoproducts [8] and efficient repair [7,8,9,10,11].

The UV spectrum is broken into three subdivisions: UV-A (315–400 nm), UV-B (280–315 nm), and UV-C (<280 nm), with UV-C being the most damaging for DNA [43] due to the shorter wavelengths. The solar spectrum (UV A/B) peaks at 300 nm and results in CPDs as the principal type of UV-lesions, placing less emphasis on 6-4PPs [27]. UV-C light is sometimes used in laboratory experiments to amplify DNA damage detection [6,24], but it does not have real world consequence for biological systems since it is absorbed by the oxygen and ozone in the Earth’s atmosphere [44].

The photoreactivity of a specific bipyrimidine pair may vary depending on the wavelength of UV light utilized, the flanking sequence conditions, and the cellular environment. For example, in one study the ratio of T^T to other CPDs was greater when UV-C light was used in experiments than when UV-B was utilized [30]. This may account for the significance placed on the T^T lesions over other photoproducts in decades of laboratory experimentation. The bipyrimidine photoreactivity coefficients utilized in our *P_g_* calculations were adapted from a study that measured UV-B induced CPDs and 6-4PPs [33].

Our data clearly show that T limitation reduces T^T formation (Figure 3). However, this is an incomplete picture. We developed the *P_g_* formula (Equation 1) to further assess other bipyrimidine impacts on photoreactivity. For example, the corresponding enrichment in C nucleotides and the higher photoreactivity of the TC bipyrimidine [33] also impact the *P_g_* score for a halophilic archaeal genome. Nevertheless, the strong, negative correlation observed between *P_g_* and G + C content (Figure 5) gives evidence that T limitation facilitates a net increase in photoprotection. Note that the three most photoreactive sequences, TC, TT, and TC [32,33], are T-containing: this could explain the observed relationship between genomic photoreactivity and G + C content. It should also be noted that C^C photolesions are the most mutagenic [32], which suggests that there is more work to be done beyond photoreactivity to explore mutagenesis from solar irradiation. Finally, determination of the role that flanking sequences [30] play in the formation of photolesions could add another dimension to this work.

From a genome evolutionary perspective, if exposure to UV light was driving an organism’s genomic bipyrimidine signature, we would expect to see this adaptation in cyanobacteria, a photosynthetic group of microorganisms, and we do not (Figure 4). Conversely, we would expect members of the enterobacteriaceae group, which dwell inside higher eukaryotes protected from the sun, to be void of UV adaptations, but in fact, their photoreactivity scores are more similar to halophilic archaea than any other sample group (Figure 4). The analysis of our controls gives evidence that there is not a clear/predictive relationship between *P*_g_ and UV-resistance alone. The similarity between the enterobacteriaceae and halophilic archaea stems not from lifestyle, but instead from their relativity high G + C contents (Table 2), which have been shown to result in low *P_g_* values (Figure 5). Hence, we find no evidence in our analysis of genomic photoreactivity that UV exposure is a selective pressure for a photoprotective bipyrimidine signature.

One important outlier is *Haloquadratum walsbyi*, a dominant halophilic archaeon in salt lakes and salterns. This microorganism is an exception to the G + C-rich genomes of the other members of this group, having only 48% G + C content (Table 1) [37,38,39]. This square-shaped organism otherwise shares the same ecosystem niche as the other extreme halophiles, thriving in high UV exposure; however, it lacks the photoprotective genome signature seen in other halophilic archaea, having a *P_g_* value of 0.260, which is over 8 standard errors smaller than the sample group’s mean of 0.245 (Figure 4). Bolhuis et al. [37] noted that *H. walsbyi* has a relatively higher number (four) photolyase genes, and this could impact its ability to counteract solar DNA damage.

If the overwhelming majority of halophilic archaea have genomes with lower photoreactivity because of their high G + C contents (Table 2), then we must ask how the G + C richness evolved? Litchfield [45] pointed to the stability of genomes rich in base pairs that have more hydrogen bonds; GC pairs have three and AT pairs have two. Halophilic archaea living in high salinity (and having high intracellular cation concentrations) would benefit from DNA helices that are more tightly paired, adding stability to their molecular structure in a destabilizing environment. High C + C content has also been discussed as a hypersaline adaptation that impacts the proteome composition of these microorganisms [46]. A + T limitations result in preferences for particular (G + C-rich) amino acids over others. For example, acidic residues such as aspartic acid would be over-represented and cysteine would be under-represented. Paul et al*.* demonstrate that this signature would reduce the likelihood of helices forming in protein tertiary structure but would positively impact coil structures. Both stronger DNA hydrogen bonding and preferential codon usage (and resulting protein structure) could impact the ability of halophilic archaea to manage life in hypersaline waters.

Genomic signatures, such as bipyrimidine limitations, should be examined when painting a picture of genome evolution for any microbial community. Each related group of species in their environment over time results in a unique signature of nucleic acid and protein composition [47,48,49], and G + C content is a marker for this signature. The UV-resistance observed in halophilic archaea can certainly be attributed to a genomic strategy. However, the driver for this is not simply UV light as other evolutionary pressures, including hypersalinity, are also significant. Indeed, high G + C reduces the photoreactivity due to the specific bipyrimidine composition, but it also impacts a combination of factors including DNA, RNA and protein-level features. Life for halophilic archaea must be well-suited for the dual extremes of both UV exposure and hypersalinity.

## Figures and Tables

**Table 1:** Halophilic archaea sampled for the present study, representative of all species with full genome sequences presently available (taxid: 183963). Data were obtained from the National Center for Biotechnology Information (NCBI) database [21].

Species	Strain	G + C (%)	Size (Mb)	Genes
*Halalkalicoccus jeotgali*	B3	62.56	3.699	3717
*Halanaeroarchaeum sulfurireducens*	HSR2	62.86	2.210	2213
*Haloarcula hispanica*	N601	62.47	3.902	3825
*Haloarcula marismortui*	ATCC 43049	61.14	4.275	4226
*Haloarcula* sp.	CBA1115	61.98	4.225	4108
*Halobacterium hubeiense*	JI20-1	66.58	3.130	3189
*Halobacterium salinarum*	NRC-1	65.92	2.571	2629
*Halobacterium* sp.	DL1	66.44	3.163	3237
*Haloferax gibbonsii*	ARA6	66.24	3.918	3783
*Haloferax mediterranei*	ATCC 33500	60.26	3.905	3800
*Haloferax volcanii*	DS2	65.46	4.013	3925
*Halogeometricum borinquense*	DSM 11551	59.97	3.944	3838
*Halomicrobium mukohataei*	DSM 12286	65.51	3.332	3293
*Halopiger xanaduensis*	SH-6	65.20	4.355	4174
*Haloquadratum walsbyi*	DSM 16790	47.90	3.179	2827
*Halorhabdus tiamatea*	SARL4B	62.77	3.146	3069
*Halorhabdus utahensis*	DSM 12940	62.90	3.117	2969
*Halorubrum lacusprofundi*	ATCC 49239	63.95	3.693	3523
*Halostagnicola larsenii*	XH-48	60.87	4.131	3954
*Haloterrigena turkmenica*	DSM 5511	64.25	5.441	5074
*Halovivax ruber*	XH-70	64.30	3.224	3187
*Natrialba magadii*	ATCC 43099	61.03	4.444	4128
*Natrinema pellirubrum*	DSM 15624	63.96	4.354	4178
*Natrinema* sp.	J7-2	64.06	3.794	3681
*Natronobacterium gregoryi*	SP2	62.20	3.788	3720
*Natronococcus occultus*	SP4	64.63	4.314	4162
*Natronomonas moolapensis*	8.8.11	64.50	2.913	2793
*Natronomonas pharaonis*	DSM 2160	63.08	2.750	2799
*Salinarchaeum* sp.	Harcht-Bsk1	66.60	3.255	3036

**Table 2:** G + C contents (%) of all sample groups utilized in the present study (archaea taxid: 2157, *n* = 68; bacteria taxid: 2, *n* = 101, cyanobacteria taxid: 1117, *n* = 32; enterobacteriaceae taxid: 91347, *n* = 42; halophilic archaea taxid: 183963, *n* = 29). Sample means are denoted with +/− 1.96 standard errors. Data was obtained from the NCBI database [21].

Sample Group	Mean G + C Content (%)	Median (%)
(Non-Halophilic) Archaea	43.87 +/− 2.30	43.05
Bacteria	49.10 +/− 2.55	47.25
Cyanobacteria	44.21 +/− 3.08	42.65
Enterobacteriaceae	47.83 +/− 3.17	51.73
Halophilic Archaea	63.09 +/− 1.28	63.95

## Supplementary Materials

The code for the R script, “DinucleotideCounts,” can be accessed at: http://www.mdpi.com/2075-1729/6/3/37/s1 or https://github.com/jones1040/DinucleotideCounts.
